# The Efficacy and Safety of First-Line Chemotherapy in Patients With Non-small Cell Lung Cancer and Interstitial Lung Disease: A Systematic Review and Meta-Analysis

**DOI:** 10.3389/fonc.2020.01636

**Published:** 2020-09-08

**Authors:** Yanning Wang, Liyun Miao, Yuxuan Hu, Yujie Zhou

**Affiliations:** ^1^Department of Respiratory and Critical Care Medicine, Nanjing Drum Tower Hospital, Nanjing, China; ^2^School of Basic Medicine and Clinical Pharmacy, China Pharmaceutical University, Nanjing, China; ^3^School of International Pharmaceutical Business, China Pharmaceutical University, Nanjing, China

**Keywords:** non-small cell lung cancer, chemotherapy, interstitial lung disease, meta-analysis, acute exacerbation

## Abstract

**Background:** Lung cancer is a well-known comorbidity of interstitial lung disease (ILD), and the actual efficacy and safety of chemotherapy for patients with non-small cell lung cancer and interstitial lung disease (NSCLC-ILD) have not been determined. We conducted this meta-analysis to assess the efficacy and safety of chemotherapy for patients with NSCLC-ILD.

**Methods:** We searched related studies from the Cochrane Library, PubMed, and Embase. The endpoints were objective response rate (ORR), disease control rate (DCR), 1-year overall survival rate (1-yOS rate), and first-line chemotherapy-related acute exacerbation of interstitial lung disease rate (AE-ILD rate).

**Results:** We included 21 studies involving 684 patients in our analysis. The pooled ORR was 43% (95% CI: 38.0–49.0%), and the pooled DCR was 80.0% (95% CI: 75.7–83.9%). The modified overall 1-yOS rate was 33.0% (95% CI: 29.0–37.0%). The pooled AE-ILD rate was 8.07% (95% CI: 6.12–10.26%). Subgroup analysis revealed a trend for lower AE-ILD rate (4.98%; 95% CI: 2.44–8.37%) in patients with carboplatin plus nab-paclitaxel. Lung function and AE-ILD may be associated with the prognosis of patients with NSCLC-ILD.

**Conclusions:** First-line chemotherapy is effective in patients with NSCLC-ILD, and the AE-ILD rate is acceptable, but the prognosis is limited. Future randomized controlled trials are needed to explore more appropriate treatment regimens to improve the prognosis of patients with NSCLC-ILD.

## Introduction

Interstitial lung disease (ILD) is a disease with progressive deterioration of respiratory function (1). ILD is an independent risk factor for lung cancer (LC). Among patients diagnosed with lung cancer, 5.8–15.2% of patients have ILD (2, 3). Epidemiological studies have shown that 22% of patients with ILD will eventually develop lung cancer; the risk is five times that of the general population (4). Recently, a study demonstrated that ILD and lung cancer share a common pathogenic mechanism (5).

In recent years, new therapies for non-small cell lung cancer (NSCLC) have developed rapidly, such as targeted therapies and immunotherapy, which can significantly prolong progression-free survival (PFS) and overall survival (OS) of patients. However, these treatment regimens are associated with a higher ILD rates in NSCLC patients with pre-existing ILD (6–9), and chemotherapy can be an appropriate option. Therefore, traditional chemotherapy still plays a critical role in first-line treatment for patients with NSCLC-ILD.

Pre-existing ILD is an independent risk factor for drug-related ILD (10). Study shows that first-line chemotherapy has a 20% incidence of acute exacerbation of interstitial lung disease (AE-ILD) in patients with NSCLC-ILD (11). The existence of ILD has previously been the exclusion criterion for most NSCLC clinical trials, and there is currently insufficient evidence to assess the risk and benefit of chemotherapy in this group of patients. In a meta-analysis conducted by Chen et al. (12), first-line chemotherapy may be associated with a higher rate of AE-ILD, and the pooled AE-ILD rate was 8.47% (95% CI: 5.04–12.6%). The overall response rate (ORR) and disease control rate (DCR) were similar to NSCLC patients without ILD, and the prognosis was slightly worse. However, in this study, 92.8% patient data were extracted from retrospective studies. More high-quality evidence is needed to support chemotherapy for NSCLC-ILD.

Recently, several multicenter prospective clinical trials (13–16) focusing on NSCLC patients with ILD have been published, and their results can improve the quality of evidence. We therefore conducted a meta-analysis to evaluate the efficacy and safety of chemotherapy in the first-line treatment of patients with NSCLC-ILD.

## Patients and Methods

### Search Strategy

We followed PRISMA guidelines for this meta-analysis (17). We searched the Cochrane Library, PubMed, and Embase for prospective studies and retrospective studies without year and language restrictions, by using the following keywords: chemotherapy, interstitial lung disease, pulmonary fibrosis, non-small cell lung cancer, and NSCLC. We also searched references of the main research and systematic reviews. The last search was updated in February 2020. Details of the search strategy are displayed in Supplemental Table 1.

### Study Selection

We included all prospective clinical trials and retrospective studies that evaluated the administration of chemotherapy as first-line treatment for patients with NSCLC-ILD. We included studies in which patients have not received systemic treatment or patients completed postoperative chemotherapy at least 1 year before recurrence.

We excluded conference abstracts and studies without sufficient data. Two investigators (YW, LM) independently reviewed the studies to select the relevant studies, and any disagreement was resolved with the consensus of the third investigator (YZ).

### Data Extraction

For each study, two investigators (YW, LM) independently extracted the following data: the year of publication, first author, study design, treatment regimens, number of patients, sex, age, score of PS, stage of disease, type of histology (squamous cell carcinoma, adenocarcinoma), ILD pattern, and data on outcome measures. The third author (YZ) assessed the data and resolved the disagreement.

### Risk of Bias and Publication Bias Assessment

Prospective clinical studies were evaluated using Methodological Index for Non-randomized Studies (MINORS) (18). MINORS developed eight items for non-comparative studies and each with a maximum score of 2. Retrospective studies were assessed using the Methods Guide for Comparative Effectiveness Reviews of the US Agency for Healthcare Research and Quality (AHRQ) (19). According to the researchers' judgments, the risk of bias for each article was classified as “low,” “high,” or “unclear.” Two investigators (YW, LM) independently assessed the risk of bias.

Publication bias was assessed with the funnel plots.

### Outcome Measures

The incidence of AE-ILD related to first-line chemotherapy was safety outcome. The treatment response [objective response rate (ORR), disease control rate (DCR)] was efficacy outcomes. The 1-year overall survival rate (1-yOS rate) was survival outcome.

### Data Synthesis and Analysis

STATA 15.0 was used to conduct a meta-analysis of the single-arm study and calculate the corresponding rates and standard errors. The pooled ES were calculated by direct equal-weighted sum method and the double arcsine method (20), and the point estimates and 95% confidence intervals (CIs) were given for each ES. The specific merging methods were as follows: If the incidence rate of most events is between [0.3, 0.7], the analysis results of the double arcsine method and the direct equal-weighted sum method are similar; if there are a considerable number of rates that are relatively large (0.8 < p < 1.0) or relatively small (0 < p < 0.2), the double arcsine method is more appropriate. In this study, most ORR values and 1-yOS rates were between 0.3 and 0.7, whereas the incidence of AE-ILD was mostly between 0 and 0.2, and the DCR mainly was between 0.8 and 1.0. Therefore, the ORR and 1-yOS rate were calculated using the direct equal-weighted sum method, and the AE-ILD rate and DCR were calculated using the double arcsine method. The heterogeneity across studies was examined by χ^2^-test and *I*^2^ statistics, and *p* < 0.05 indicated significant heterogeneity. Studies with *I*^2^ statistics of 25–50%, 50–75%, and >75% were considered to have low, moderate, and high heterogeneity, respectively (21). Considering that there might exist subjectivity due to the lack of control groups, all ES were pooled by random-effects models (22). Subgroup analyses were conducted according to the treatment regimen for ORR, 1-yOS, and AE-ILD. The difference between each subgroup was judged by the *Z*-test, and *p* < 0.05 indicating statistical significance.

## Results

### Study Selection

**Figure 1:**
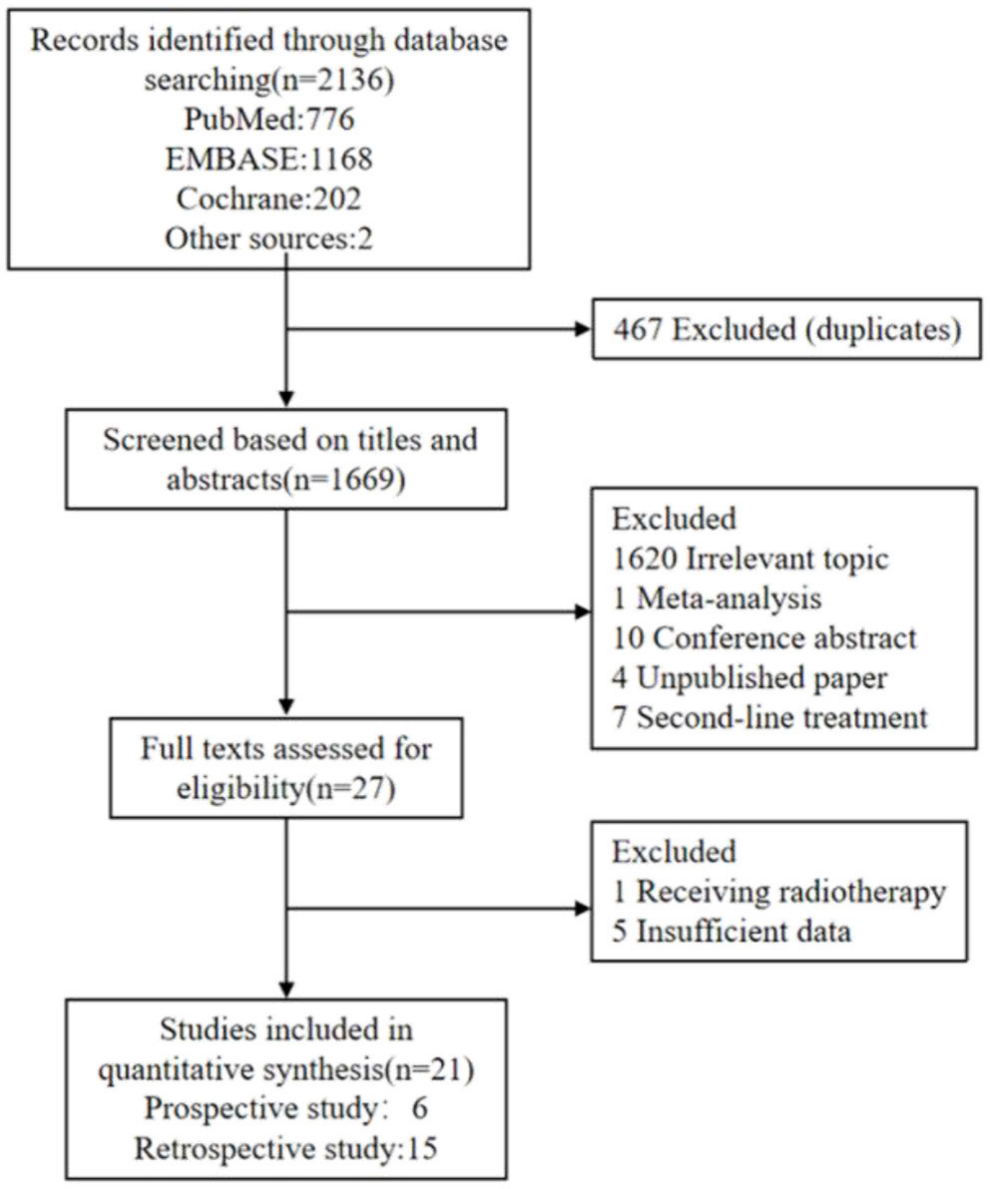
Flowchart diagram of study selection.

A total of 2,136 records were retrieved by initial search from the Cochrane Library, PubMed, and Embase. Based on duplicate records and the review of titles or abstracts, 2,109 articles were excluded, resulting in 27 potentially eligible studies. Finally, 6 prospective clinical studies and 15 retrospective clinical studies, published from 2010 to 2019, were included in the meta-analysis (Figure 1).

### Trial Characteristics

The characteristics of each study are shown in Table 1. The 21 eligible studies (13–16, 23–39) included 684 patients. Patients in each study had similar average age and sex ratio. Of the 21 studies, 8 studies (15, 23, 26, 28, 29, 32, 33, 38) mainly administered carboplatin + paclitaxel, 7 studies (13, 14, 25, 30, 34, 35, 37) administered carboplatin + nab-paclitaxel, 2 studies (16, 24) administered carboplatin + S-1, 1 study (27) administered cisplatin + vinorelbine, 1 study (31) administered cisplatin + etoposide, 1 study (36) administered platinum + pemetrexed, and 1 study (39) administered carboplatin + pemetrexed/gemcitabine. Six prospective studies (13–16, 23, 24) included 237 patients. Among these patients, most patients were with good performance status (PS 0–1). The clinical stages were mainly IIIA, IIIB, and IV or recurrence after surgery. Fifteen retrospective studies (25–39) included 447 patients. Among these patients, most patients were with PS of 0–2. The clinical stages were mainly IIIA, IIIB, and IV.

**Table 1:** Characteristics of eligible studies.

**Authors, year**	**Study design**	**Treatment**	**Patients enrolled**	**Mean age, years**	**Male, *n* (%)**	**Current/former smokers, *n* (%)**	**Stage (*n*)**	**Histology (*n*), Ad/Sq/others**	**ILD pattern (*n*), UIP/non-UIP**	**Lung function (%),median FVC/DLco**	**Total AE-ILD rate (%)**	**PS (*n*)**
Kenmotsu et al. (13)	Phase II	CB + nab-PTX	94	70	89		IIIA/IIIB/IV/Recurrent (15/23/47/9)	49/39/6	50/44	90.1/63.7	20.7	0/1 (42/52)
Asahina et al. (14)	Phase II	CB + nab-PTX	36	68.5	72.2	97.2	IIIB/IV/Recurrent (15/18/3)	16/15/5	12/24	96.4/73.1	11.1	0/1 (13/23)
Minegishi et al. (23)	Pilot	CB + weekly PTX	18	71	77.8	83.3	IIIA/IIIB/IV or Recurrent (2/3/13)	6/7/5	6/12	82/–	27.8	0/1 (7/11)
Fukuizumi et al. (15)	Phase II	CB + weekly PTX	35	68	88.6	94.3	IIIA/IIIB/IV/Recurrent (15/7/10/3)	13/16/6	18/17	89/70	18.2	0/1 (18/17)
Sekine et al. (24)	Pilot	CB + S-1	21	67	90.5	95.2	IIB/IIIIA/IIIB/IV/Recurrent (1/2/10/4/4)	10/10/1	12/9	91/63.4	38.1	0/1/2 (7/12/2)
Hanibuchi et al. (16)	Phase II	CB + S-1	33	70	90.9	90.9	IIIB/IV/Recurrent (7/19/7)	13/16/4	22/11			0/1 (18/15)
Yasuda et al. (25)	Retrospective	CB + nab-PTX	12	73	91.7	100	IIIA/IIIB/IV (1/4/7)	7/4/1	3/9	81.7/90.7	8.3	0–1/ 2(11/1)
Watanabe et al. (26)	Retrospective	CB + weekly PTX CB + DOC NVB	21	68.4	85.7	100	IIIB/IV (11/10)	14/5/2	18/3	91.6/51.2		0/1/2 (8/10/3)
Watanabe et al. (27)	Retrospective	DDP + NVB	67	64	95.5	100	IIIB/IV/Recurrent (20/42/5)	26/21/20				0/1/2 (11/53/3)
Shukuya et al. (28)	Retrospective	CB + weekly PTX	15	68	86.7		IIIA/IIIB/IV/Recurrent (1/5/7/2)	10/5/0	4/11			0/1/2 (6/7/2)
Kinoshita et al. (29)	Retrospective	CB + weekly PTX DDP + NVB DDP + DOC	22	70	95.5	100	IIIA/IIIB/IV or Recurrent (1/6/15)	11/7/4		87.2/–		0/1 (12/10)
Igawa et al. (30)	Retrospective	CB + nab-PTX	34	71	85	97	IIIA/IIIB/IV or Recurrent (2/2/30)	12/16/6	16/18			0–1/2–3 (32/2)
Yamaguchi et al. (31)	Retrospective	DDP + VP-16	24	63	95.8		IIIA/IIIB/IV (5/6/13)	12/4/8	20/4		20.8	0/1/2 (14/8/2)
Kenmotsu et al. (32)	Retrospective	Platinum-based chemotherapy	104	67	91.3	100	IIIA or IIIB/IV/Recurrent (41/55/8)	50/47/7	70/34		25	0–1/2 (96/8)
Shimizu et al. (33)	Retrospective	CB + weekly PTX	11	72	91	91	IIIA/IIIB/IV (2/2/7)	11/0/0	3/8			0 or 1
Araya et al. (34)	Retrospective	CB + nab-PTX	9	69	88.9	100	IIIB/IV (2/7)	1/7/1	5/4	112.4/55.8	22.2	0–1/2 (7/2)
Niwa et al. (35)	Retrospective	CB + nab-PTX	9	67	100	100	IIIA/IIIB/IV (1/1/7)	0/6/3	6/3			0/1/2 (0/8/1)
Fujita et al. (36)	Retrospective	PT + PEM	24	70	91.7	95.8	IIIA or IIIB/IV or Recurrent (8/16)	24/0/0	2/22	91.2/–	41.7	0–1/2 (22/2)
Fujita et al. (37)	Retrospective	CB + nab-PTX	8	77	87.5	100	IIIA/IIIB/IV (5/1/2)	0/8/0	4/4	81.5/–	25	0/1 (3/5)
Kakiuchi et al. (38)	Retrospective	Platinum-based chemotherapy	35	72	93.2	98.6		15/20/0				
Choiet al. (39)	Retrospective	CB + GEM CB + PEM	52	67	86.5	86.5	I/II/III/IV (2/2/11/37)	32/13/7			13.5	0–1/2 (47/5)

*PS, performance status; CB, carboplatin; DDP, cisplatin; PT, platinum; nab-PTX, nano albumin paclitaxel; PTX, paclitaxel; S-1, tegafur-gimeracil-oteracil potassium; DOC, docetaxel; NVB, vinorelbine; VP-16, etoposide; PEM, pemetrexed; GEM, gemcitabine; Sq, squamous; Ad, adenocarcinoma; FVC, forced vital capacity; DLco, diffusing capacity of the lungs for carbon monoxide; UIP, usual interstitial pneumonia*.

### Quality and Publication Bias Assessment

All studies used intention-to-treat (ITT) analysis to handle missing data. No study used blind methods for patient intervention and exposure status (Supplemental Tables 2, 3). Most retrospective studies had high risk of biases, mostly regarding selection bias and the absence of confounding variable assessment.

No evidence of publication bias was detected by funnel plot (Supplemental Figure 1).

### Tumor Response

**Figure 2:**
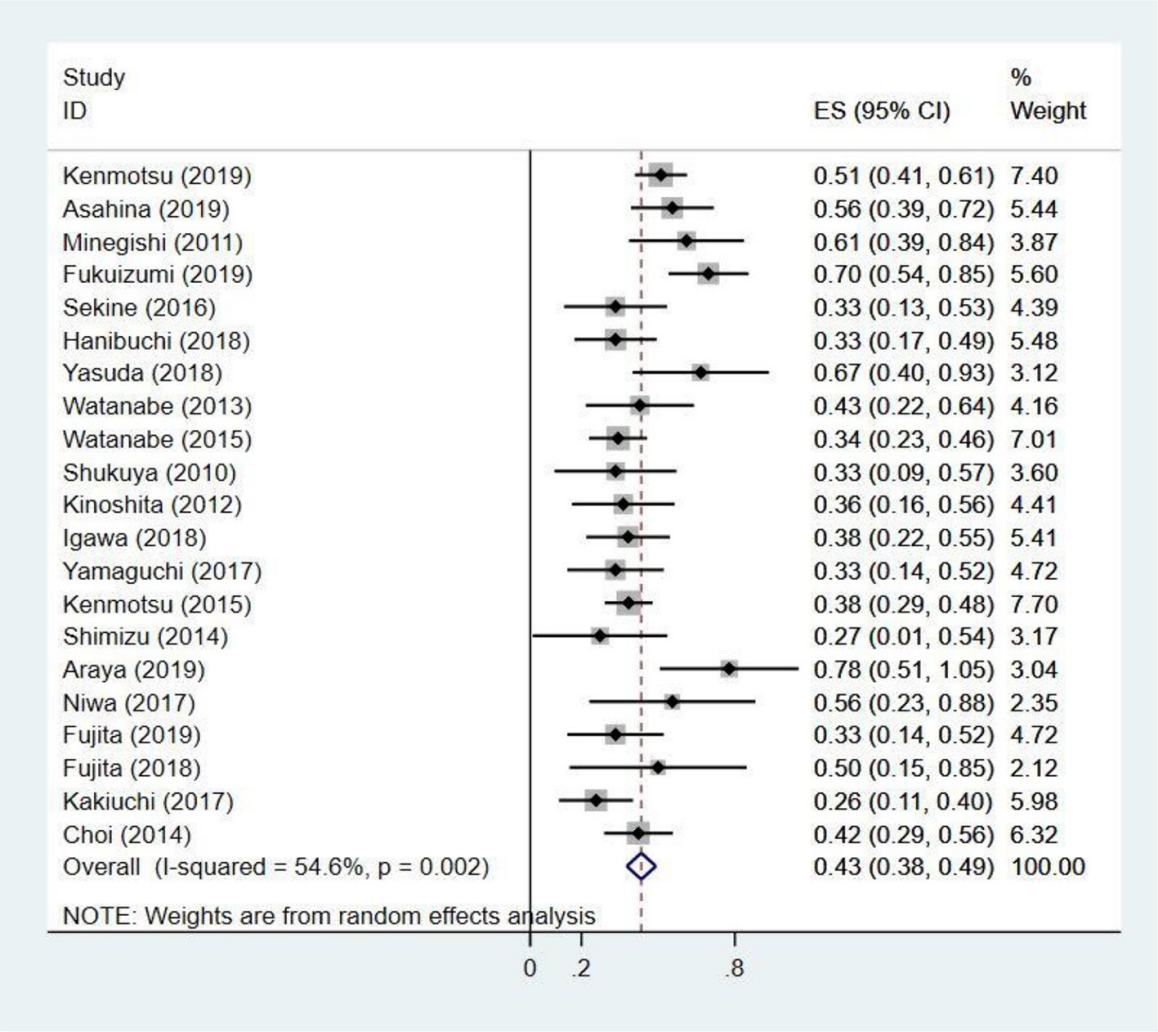
The pooled objective response rate of chemotherapy for patients with non-small cell lung cancer and interstitial lung disease.

The ORR ranged from 27 to 78%, extracted from 21 studies (13–16, 23–39) including 684 patients. Adopting random-effects model, the pooled ORR was 43% (95% CI: 38.0–49.0%) with moderate heterogeneity (*I*^2^ = 54.6%, *p* = 0.002) (Figure 2). The results of subgroup analysis suggested that ORR had obvious association with treatment regimen (*p*-value for subgroup difference = 0.023) (Supplemental Figure 2) and median follow-up time (*p* = 0.017) (Supplemental Table 5).

The DCR ranged from 31 to 98%, extracted from 20 studies (13–16, 23–37, 39) with 649 patients. Adopting random-effects model, the pooled DCR was 80.0% (95% CI: 75.7–83.9%) with low heterogeneity (*I*^2^ = 37.3%, *p* = 0.048) (Supplemental Figure 3).

### Overall Survival

**Figure 3:**
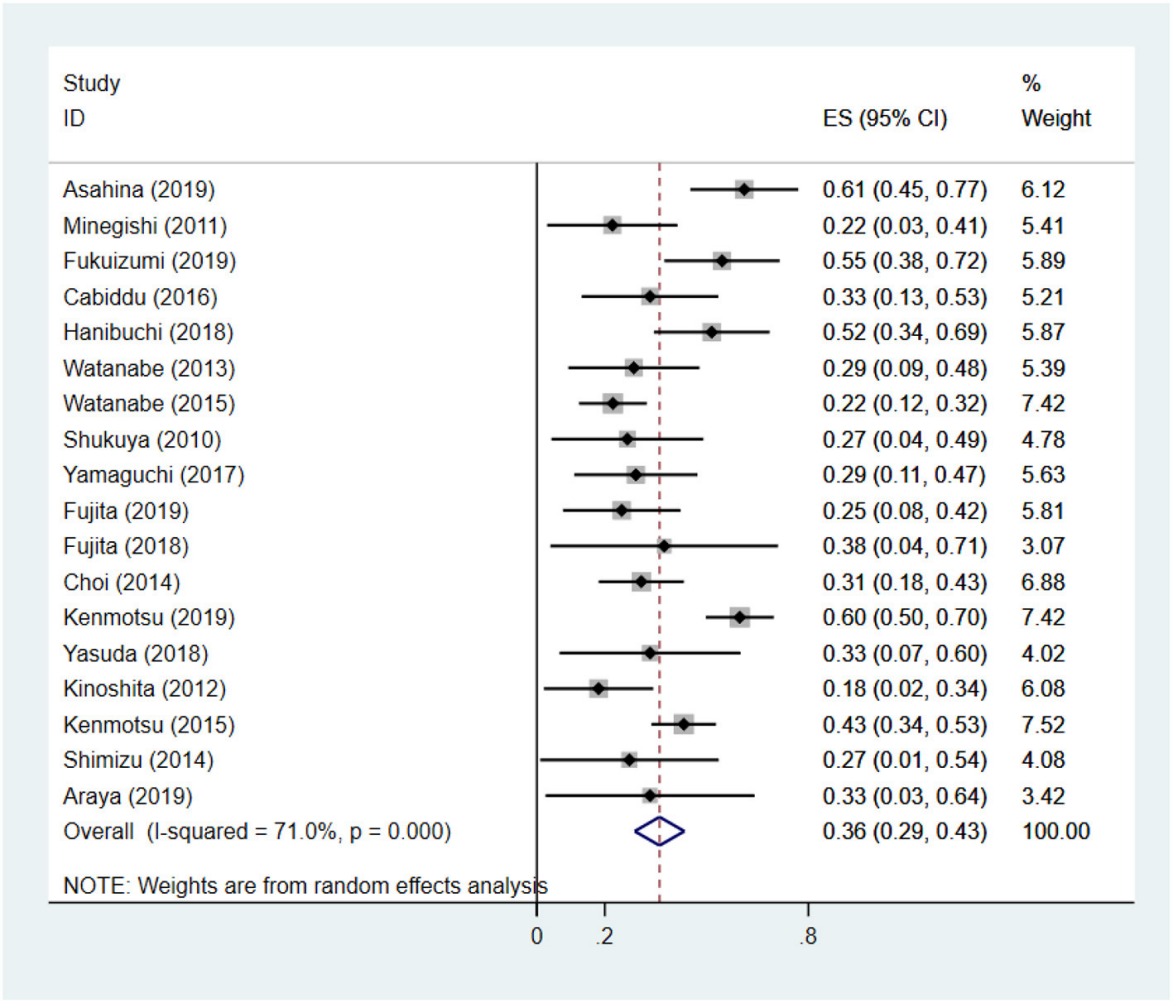
The pooled 1-year overall survival rate of chemotherapy for patients with non-small cell lung cancer and interstitial lung disease.

The 1-yOS rate ranged from 18 to 61%, extracted from 18 studies (13–16, 23–34, 36, 37, 39) including 602 patients. Adopting random-effects model, the 1-yOS rate was 36.1% (95% CI: 28.8–43.5%) with moderate heterogeneity (*I*^2^ = 71.0%, *p* < 0.001) (Figure 3). After removing two studies with high heterogeneity (13, 14), the modified overall 1-yOS rate was 33.0% (95% CI: 29.0–37.0%) with low heterogeneity (*I*^2^ = 41.9%, *p* = 0.040) (Supplemental Figure 4).

To explore the heterogeneity of the 1-yOS rate, we use the following categorical variables for subgroup analysis: treatment regimens (CB + nab-PTX, CB + PTX, CB + S-1, other treatment regimens), median follow-up time (<10 months, ≥10 months), total AE-ILD rate (<25%, ≥25%), and lung function (median FVC >85% and median DLco >60%, median FVC ≤ 85% or median DLco ≤ 60%). The results are displayed in Table 2. Regarding heterogeneity, treatment regimens, total AE-ILD rate, lung function, and median follow-up time were likely to be the main sources of heterogeneity.

**Table 2:** Subgroup analysis of 1-year overall survival rate of chemotherapy for patients with non-small cell lung cancer and interstitial lung disease.

**Group**	**Studies (*n*)**	**N**	**ES (95% CI)**	***P*-values**	***I*^**2**^ (%)**
**Treatment regimens**					
CB + nab-PTX	5	157	0.45 (0.32, 0.59)	<0.001	58.9
CB + PTX	4	77	0.34 (0.17, 0.50)		60.7
CB + S-1	2	54	0.43 (0.26, 0.61)		45.1
Other treatment regimens	4	167	0.26 (0.19, 0.33)		29.8
**Median follow-up time, months**					
<10	4	124	0.23 (0.16, 0.30)	<0.001	0.0
≥10	4	213	0.51 (0.45, 0.58)		79.3
**Total AE-ILD rate, %**					
<25	7	258	0.48 (0.42, 0.53)	<0.001	72.6
≥25	5	175	0.36 (0.29, 0.43)		31.5
**Lung function**					
Median FVC > 85% and median DLco > 60%	4	182	0.56 (0.49, 0.63)	<0.001	48.5
Median FVC ≤ 85% or median DLco ≤ 60%	5	68	0.29 (0.18, 0.40)		0.0

*N, number of patients; ES, effect size; CB, carboplatin; nab-PTX, nano albumin paclitaxel; PTX, paclitaxel; S-1, tegafur-gimeracil-oteracil potassium; FVC, forced vital capacity; DLco, diffusing capacity of the lungs for carbon monoxide*.

*Other treatment regimens: cisplatin + vinorelbine; cisplatin + etoposide; platinum + pemetrexed; carboplatin + gemcitabine*.

### Acute Exacerbation of Interstitial Lung Disease

**Figure 4:**
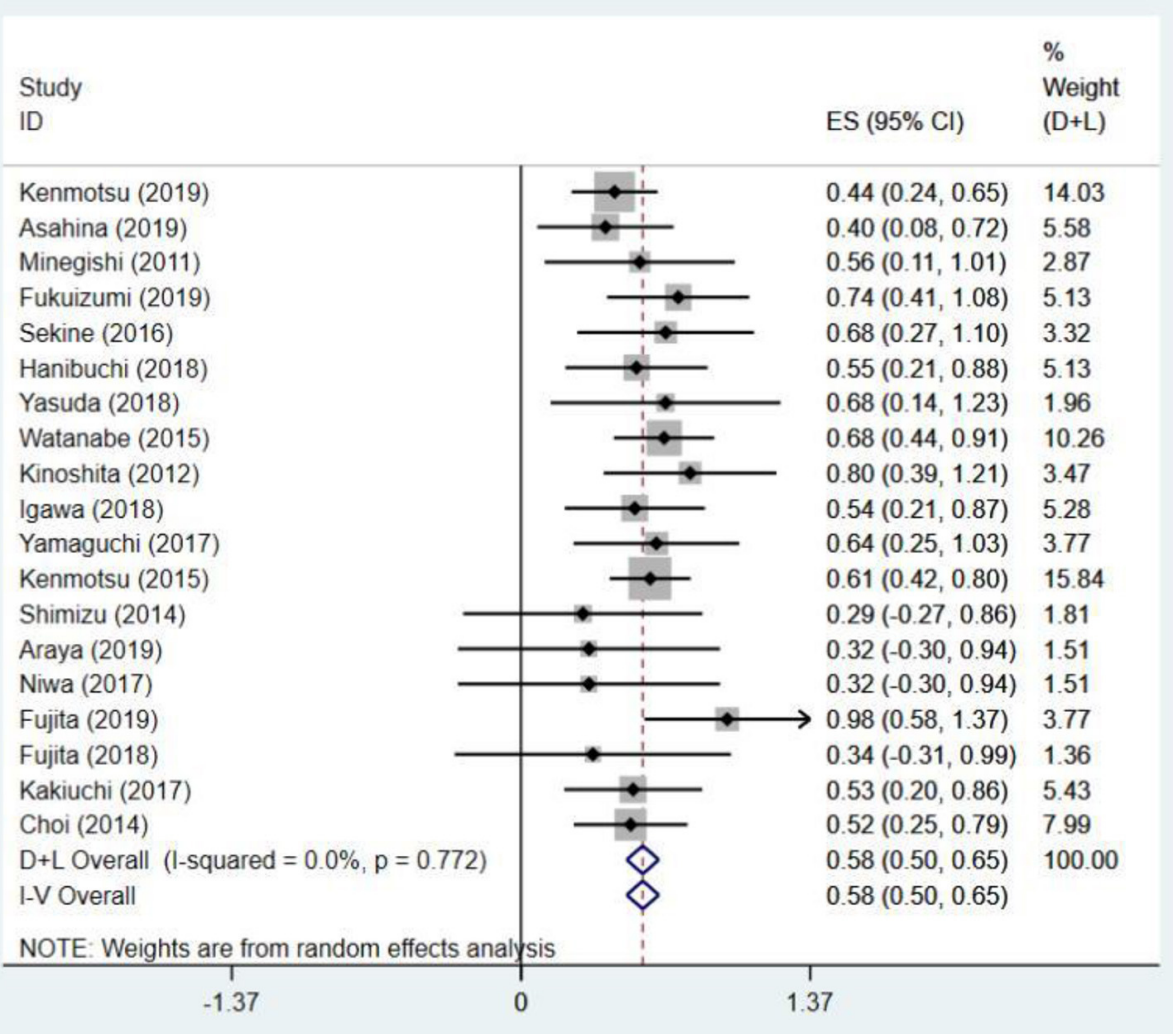
The pooled first-line chemotherapy-related acute exacerbation of interstitial lung disease rate for patients with non-small cell lung cancer and interstitial lung disease.

Analyzed from the data of 644 patients in 19 studies (13–16, 23–25, 27, 29–39), the AE-ILD rate ranged from 2.8 to 20.8%. The pooled AE-ILD rate was 8.07% (95% CI: 6.12–10.26%) with no heterogeneity (*I*^2^ = 0.0%, *p* = 0.772) (Figure 4). The stratification of treatment regimen showed potential differences in AE-ILD rate (*p*-value for subgroup difference = 0.093) (Supplemental Figure 5). The nab-PTX group had lower AE-ILD rate than the other treatment regimens group (4.98 vs. 11.92%, *p* = 0.018), whereas no statistical differences between other groups were noted (Supplemental Table 4). Two studies (26, 28) were excluded because the diagnostic criteria for AE-ILD were not given, and the definition of AE-ILD was different from other studies.

## Discussion

Our study has implications for future research on the treatment and management of patients with NSCLC-ILD. Chemotherapy is still the main treatment regimen for NSCLC patients with pre-existing ILD. A series of studies (13–16, 23–39) have investigated the risk and benefit of chemotherapy for NSCLC-ILD; however, all of these studies are single-arm trials, and part of them have a small sample size. Therefore, it is necessary to summarize the existing data to provide a basis for future clinical studies.

First line of platinum-based chemotherapy for advanced NSCLC has an ORR of 25–33%, 1-yOS rate of 41.9% (40, 41). In our study, the ORR, DCR, and 1-yOS rates were 43, 80, and 33%, respectively. The tumor response was superior to NSCLC patients without ILD, whereas 1-yOS rate was poor (*p* < 0.05) (Supplemental Table 6). We found the 1-yOS rate across the studies varied from 18 to 61% with significant heterogeneity. Different study design may contribute to this heterogeneity. We hence undertook subgroup analysis. Subgroup analysis demonstrated treatment regimens, lung function, and total AE-ILD rate had a significant correlation with 1-yOS rate. It is worth noting that several prospective studies had significantly higher 1-yOS rates than retrospective studies (13–16), ranging from 51.4 to 61.1%. We found that patients included in the prospective studies had better median lung function status than patients in the retrospective studies (Table 1), and our subgroup analysis showed the difference in 1-yOS rate between studies with median lung function better and poorer was significant (56.0 vs. 29.0%, *p* < 0.001) (Table 2). In the real-world population, composed of unselected NSCLC-ILD patients, almost always afflicted by more severe lung function limitation and respiratory impairment, our results emphasized the importance of evaluating the impact of lung function on the prognosis of chemotherapy for patients with NSCLC-ILD. Considering the retrospective studies included in our analysis have selection bias, and confounding factors such as the severity of ILD and the type of ILD may affect the prognosis, more researches are needed to verify our findings in the future.

The ORR was 43% with moderate heterogeneity in this study. The results of subgroup analysis showed that ORR was associated with treatment regimens, and heterogeneity was mainly generated in the paclitaxel group. After comparing six studies in the paclitaxel group, we found that patients receiving paclitaxel 100 mg/m^2^ weekly had a higher ORR (15, 23), 61 and 70%, respectively. In the remaining studies, patients received paclitaxel 60–70 mg/m^2^ weekly had poor ORR. The CA031 study (41) showed that nanoparticle albumin-bound-paclitaxel (nab-PTX) plus carboplatin produced a significantly higher ORR than solvent-based paclitaxel plus carboplatin in patients with squamous cell carcinoma (SCC) (41 vs. 24%, *p* < 0.001). In our analysis, patients with SCC accounted for a relatively high proportion, especially in the nab-PTX group (Table 1). Therefore, some studies have selection bias (34, 35, 37) and caused ORR in our analysis to be significantly higher than patients without ILD.

The incidence of AE-ILD was pooled to evaluate the safety of first-line chemotherapy in patients with NSCLC-ILD; the pooled AE-ILD rate was 8.07% (95% CI: 6.12–10.26%) with no heterogeneity (*I*^2^ = 0%, *p* = 0.772). In Chen's meta-analysis, the pooled AE-ILD rate was 8.47% (95% CI: 5.04–12.6%), which is very similar to our results. In this study, carboplatin + paclitaxel was the main treatment regimen, whereas our study included new treatment regimens such as carboplatin + nab-paclitaxel and carboplatin + S-1; this implies that the difference in the incidence of AE-ILD between treatment regimens may not be significant. A previous study (11) has shown that the incidence of AE-ILD related to chemotherapy was 20%, which is higher than our results. In this study, the definition of chemotherapy-related AE-ILD was that AE-ILD occurred within 6 months after the last chemotherapy, whereas in our study, it was defined as occurring within 1 month. Two phase III trials (42) evaluated the benefit of nintedanib in idiopathic pulmonary fibrosis (IPF) patients; the proportion of patients with AE-IPF within 1 year was 3.6–9.6%. According to these studies, the safety of first-line chemotherapy in NSCLC-ILD patients is acceptable. We performed a subgroup analysis based on treatment regimens (Supplemental Figure 5) to investigate which first-line chemotherapy regimen has the lowest risk of AE-ILD. The results showed that the risk of AE-ILD in the nab-PTX group was lower than the other treatment regimens group with statistical significance (4.98 vs. 11.92%, *p* = 0.018), and the risk of AE-ILD in the nab-PTX group also tended to be lower than the PTX group and S-1 group (Supplemental Table 4). This indicated that carboplatin plus nab-paclitaxel tends to be a safer chemotherapy regimen. However, considering that this subgroup analysis relies on a small number of patients, more data are needed to support our results in the future. Recently, a study found that AE-ILD might result in poorer prognosis in patients with lung cancer and interstitial lung disease, and AE-ILD was associated with poor prognosis only in the small cell lung cancer group, but not in the NSCLC group (43). In our study, the total AE-ILD rate ranged from 11.1 to 41.7%. The subgroup analysis based on total AE-ILD rate with 25% as the cut-off point revealed that higher AE-ILD rate was associated with a worse 1-yOS rate (48 vs. 36%, *p* = 0.013) (Table 2). A study (44) reported that first-line chemotherapy combined with bevacizumab could reduce the incidence of chemotherapy-related AE-ILD in patients with NSCLC-ILD (0 vs. 22.6%, *p* = 0.037), At the same time, a phase II trial (45) that examines whether nintedanib combined with carboplatin plus nab-paclitaxel can prolong the interval to acute exacerbation of IPF is underway. In the future, those studies may provide new options for the treatment of patients with NSCLC-ILD.

Most current studies only focus on the first-line treatment regimens for patients with NSCLC-ILD, and rarely explore optimal second-line treatment regimen. Second-line chemotherapy for patients with NSCLC-ILD has worse efficacy and higher risk of AE-ILD than first-line chemotherapy. Some retrospective studies (46) found that the risk of second-line pemetrexed-related AE-ILD ranged from 12.0 to 18.1%. Watanabe et al. (47) reported that in NSCLC-ILD patients treated with docetaxel monotherapy in a second-line setting, the ORR, DCR, OS, and incidence of AE-ILD were 8.6%, 37.1%, 5.1 months, and 14.3%, respectively. These studies demonstrated that second-line docetaxel and pemetrexed single-agent chemotherapy have poor efficacy and high risk in NSCLC-ILD patients. Novel treatment regimens should be explored in this setting. In a study performed by Fukuizumi et al. (15), 12 patients received second-line treatment with paclitaxel containing regimen again, the ORR of this challenge group was 50%, and there were no cases of AE-ILD. In contrast, Kakiuchi et al. (14) found the risk of AE-ILD with paclitaxel in the later lines of treatment was twice that of first-line treatment and reached 15.2%, whereas no AE-ILD associated with S-1 was observed in the later lines of treatment. Recently, a multicenter phase II trial conducted by Fujimoto et al. (48) demonstrated that nivolumab is a potential option for previously treated NSCLC patients with mild ILD. The study enrolled 18 NSCLC patients with ILD who have relatively good pulmonary function and less severe interstitial pneumonia pattern on CT (mild ILD). The ORR, DCR, and 6-month PFS rate were 39, 72, and 56%, respectively. Two patients (11%) had grade 2 pneumonitis which improved with corticosteroid therapy. More studies should be conducted in the future to explore optimal second-line treatment regimen to improve the prognosis of patients with NSCLC-ILD.

This meta-analysis has several limitations. First, the included studies were all single-arm clinical studies; none of the included studies were randomized controlled trials (RCTs). Therefore, we only evaluated the efficacy and risk without definite conclusions. However, the single-arm trial design is of great significance for exploring NSCLC-ILD treatment options because there is currently no standard treatment option, and this study could provide the basis for further research in this field. Second, we included many retrospective studies that may produce selective bias, and more high-quality studies are needed to verify our results in the future. Third, three studies had small sample sizes (34, 35, 37) and might cause heterogeneity. However, no statistical heterogeneity was observed. Finally, all the studies we included were performed in Japan; therefore, the extrapolation of the results might be limited.

## Conclusions

In summary, our study indicated that first-line chemotherapy is effective in patients with NSCLC-ILD, and the incidence of AE-ILD is acceptable, but the prognosis is limited. Lung function and AE-ILD may be related to the prognosis of patients with NSCLC-ILD. Compared with other treatment regimens, carboplatin plus nab-paclitaxel regimen tends to have a lower incidence of AE-ILD. Future well-designed RCTs are required to explore new treatment options to improve the prognosis of patients with NSCLC-ILD.

## Data Availability Statement

All datasets generated for this study are included in the article/Supplementary Material.

## Author Contributions

YZ: writing- reviewing, editing, and conceptualization. LM: writing- reviewing, editing, and supervision. YW: writing- original draft preparation, investigation conducting a research, and investigation process. YH: software and data curation. All authors contributed to the article and approved the submitted version.

## Conflict of Interest

The authors declare that the research was conducted in the absence of any commercial or financial relationships that could be construed as a potential conflict of interest.

## Abbreviations

LC: lung cancer
NSCLC: non-small cell lung cancer
ILD: interstitial lung disease
NSCLC-ILD: non-small cell lung cancer and interstitial lung disease
AE: acute exacerbation
AE-ILD: acute exacerbation of interstitial lung disease
ORR: overall response rate
DCR: disease control rate
1-yOS: 1-year overall survival rate
PFS: progression-free survival
OS: overall survival
CI: confidence interval
RCTs: randomized controlled trials
PS: performance status
CBDCA: carboplatin
DDP: cisplatin
PT: platinum
nab-PTX: nano albumin paclitaxel
PTX: paclitaxel
S-1: tegafur-gimeracil-oteracil potassium
DOC: docetaxel
NVB: vinorelbine
VP-16: etoposide
PEM: pemetrexed
GEM: gemcitabine
ITT: intention-to-treat.
