# Supplementary material for: The Efficacy and Safety of First-Line Chemotherapy in Patients With Non-small Cell Lung Cancer and Interstitial Lung Disease: A Systematic Review and Meta-Analysis
Source: Front Oncol. 2020 Sep 8;10:1636. doi: 10.3389/fonc.2020.01636 (PMC7506119; doi:10.3389/fonc.2020.01636)

**Supplementary material**

**Supplemental Table 1. The search strategy of Pubmed.**

((((((((((((((((((platinum based agent) OR cisplatin) OR carboplatin) OR gemcitabine) OR vinorelbine) OR ifosfamide) OR etoposide) OR irinotecan) OR vinblastine) OR docetaxel) OR taxotere) OR pemetrexed) OR paclitaxel) OR taxanes) OR mitomycin) OR chemotherapy)) AND ((((((((((* Interstitial Lung Disease) OR * Interstitial Lung Diseases) OR * Diffuse Parenchymal Lung Diseases) OR * Interstitial Pneumonia) OR * Interstitial Pneumonias) OR * Interstitial Pneumonitides) OR * Interstitial Pneumonitis)) OR "Lung Diseases, Interstitial"[Mesh]) OR (((((Pulmonary Fibrosis) OR * Pulmonary Fibroses) OR * Fibrosing Alveolitides) OR * Fibrosing Alveolitis) OR * Idiopathic Diffuse Interstitial Pulmonary Fibrosis))) AND ((((((((((((Carcinoma, Non Small Cell Lung) OR Carcinomas, Non-Small-Cell Lung) OR Lung Carcinoma, Non-Small-Cell) OR Lung Carcinomas, Non-Small-Cell) OR Non-Small-Cell Lung Carcinomas) OR Nonsmall Cell Lung Cancer) OR Non-Small-Cell Lung Carcinoma) OR Non Small Cell Lung Carcinoma) OR Carcinoma, Non-Small Cell Lung) OR Non-Small Cell Lung Cancer)) OR "Carcinoma, Non-Small-Cell Lung"[Mesh])

**Supplemental Table 2. Risk of bias summary of 6 prospective clinical studies**

| **Study** | Kenmotsu 2019 | Asahina 2019 | Minegishi 2011 | Fukuizumi 2019 | Cabiddu 2016 | Hanibuchi 2018 |
| --- | --- | --- | --- | --- | --- | --- |
| **A clearly stated aim** | 2 | 2 | 2 | 2 | 2 | 2 |
| **Inclusion of consecutive patients** | 2 | 2 | 2 | 2 | 2 | 2 |
| **Prospective collection of data** | 2 | 2 | 2 | 2 | 2 | 2 |
| **Endpoints appropriate to the aim of the study** | 2 | 2 | 2 | 2 | 2 | 2 |
| **Unbiased assessment of the study endpoint** | 0 | 0 | 0 | 0 | 0 | 0 |
| **Follow-up period appropriate to the aim of the study** | 2 | 2 | 2 | 2 | 2 | 2 |
| **Loss to follow up less than 5%** | 2 | 2 | 2 | 2 | 2 | 2 |
| **Prospective calculation of the study size** | 2 | 2 | 1 | 2 | 2 | 2 |
| **Score** | 14 | 14 | 13 | 14 | 14 | 14 |

The items are scored 0 (not reported), 1 (reported but inadequate) or 2 (reported and adequate). The global ideal score being 16 for non-comparative studies and 24 for comparative studies.

| **Study** | **Selection bias** | **Performance bias** | | **Attrition bias** | **Detection bias** | | | | **Reporting bias** |  |  |  |  |  |  |  |
| --- | --- | --- | --- | --- | --- | --- | --- | --- | --- | --- | --- | --- | --- | --- | --- | --- |
|  | 1 | 2 | 3 | 4 | 5 | 6 | 7 | 8 | 9 |  |  |  |  |  |  |  |
| Yasuda 2018 | high | low | low | low | high | low | low | high | low |  |  |  |  |  |  |  |
| Watanabe 2013 | high | low | low | low | high | low | low | high | low |  |  |  |  |  |  |  |
| Watanabe 2015 | high | low | low | low | high | low | low | high | low |  |  |  |  |  |  |  |
| Shukuya 2010 | high | low | low | low | high | low | low | high | low |  |  |  |  |  |  |  |
| Kinoshita 2012 | low | low | low | low | high | low | low | low | low |  |  |  |  |  |  |  |
| Igawa 2018 | high | low | low | low | high | low | low | high | low |  |  |  |  |  |  |  |
| Yamaguchi 2017 | high | low | low | low | high | low | low | high | low |  |  |  |  |  |  |  |
| Kenmotsu 2015 | low | low | low | low | high | low | low | low | low |  |  |  |  |  |  |  |
| Shimizu 2014 | low | low | low | low | high | low | low | high | low |  |  |  |  |  |  |  |
| Araya 2019 | high | low | low | low | high | low | low | high | low |  |  |  |  |  |  |  |
| Niwa 2017 | high | low | low | low | high | low | low | high | low |  |  |  |  |  |  |  |
| Fujita 2019 | high | low | low | low | high | low | low | high | low |  |  |  |  |  |  |  |
| Fujita 2018 | high | low | low | low | high | low | low | high | low |  |  |  |  |  |  |  |
| Kakiuchi 2017 | low | low | low | low | high | low | low | low | low |  |  |  |  |  |  |  |
| Choi 2014 | low | low | low | low | high | low | low | low | low |  |  |  |  |  |  |  |

**Supplemental Table 3. Risk of bias summary of 15 retrospective clinical studies**

1. Does the design or analysis control account for important confounding and modifying variables through matching, stratification, multivariable analysis, or other approaches?

2. Did researchers rule out any impact from a concurrent intervention or an unintended exposure that might bias results?

3. Did the study maintain fidelity to the intervention protocol?

4. If attrition (overall or differential nonresponse, dropout, loss to follow-up, or exclusion of participants) was a concern, were missing data handled appropriately (e.g., intention-to-treat analysis and imputation)?

5. Were the outcome assessors blinded to the intervention or exposure status of participants?

6. Were interventions/exposures assessed/defined using valid and reliable measures, implemented consistently across all study participants?

7. Were outcomes assessed/defined using valid and reliable measures, implemented consistently across all study participants?

8. Were confounding variables assessed using valid and reliable measures, implemented consistently across all study participants?

9. Were the potential outcomes prespecified by the researchers? Are all prespecified outcomes reported?

**Supplemental Table 4. Subgroup analysis of acute exacerbation of interstitial lung disease (AE-ILD) rate with different treatment regimens**

|  | 1 | |  | 2 | |  | 3 | |
| --- | --- | --- | --- | --- | --- | --- | --- | --- |
| **Group** | CB+nab-PTX | Other treatment regimens |  | CB+nab-PTX | CB+PTX |  | CB+nab-PTX | CB+S-1 |
| **Studies (n)** | 7 | 6 |  | 7 | 6 |  | 7 | 4 |
| **ES (95% CI)** | 4.98%  ( 2.44–8.37%) | 11.92%  (6.81-18.22%) |  | 4.98%  ( 2.44–8.37%) | 10.05%  (5.88-15.16) |  | 4.98%  ( 2.44–8.37%) | 8.45%  (3.33-15.63) |
| **I^2^ (%)** | 0.0% | 20.8% |  | 0.0% | 0.0% |  | 0.0% | 0.0% |
| **P Values** | 0.018 | |  | 0.065 | |  | 0.296 | |

Abbreviations: ES: effect size; CB: carboplatin; nab-PTX: nano albumin paclitaxel; PTX: paclitaxel; S-1: tegafur-gimeracil-oteracil potassium.

Other treatment regimens: cisplatin + vinorelbine; cisplatin + etoposide; platinum + pemetrexed; carboplatin + gemcitabine.

**Supplemental Table 5. Subgroup analysis of ORR and AE-ILD rate of chemotherapy for patients with non-small cell lung cancer and interstitial lung disease**

| **Group** | **Studies (n)** | **N** | **ES (95% CI)** | **P Values** | **I2 (%)** |
| --- | --- | --- | --- | --- | --- |
| **ORR** | | | | | |
| Median follow-up time |  |  |  |  |  |
| <10 months | 4 | 124 | 0.34 (0.26, 0.42) | 0.017 | 0.0% |
| ≥10 months | 4 | 213 | 0.47 (0.40, 0.53) |  | 39.0% |
| Lung function |  |  |  |  |  |
| median FVC>85% and median DLco>60% | 4 | 182 | 0.54 (0.47, 0.61) | 0.459 | 63.5% |
| median FVC≤85% or median DLco≤60% | 5 | 68 | 0.59 (0.47, 0.70) |  | 14.5% |
| **AE-ILD** | | | | | |
| Median follow-up time |  |  |  |  |  |
| <10 months | 4 | 124 | 0.054 (0.028, 0.088) | 0.096 | 0.0% |
| ≥10 months | 4 | 213 | 0.104 (0.057, 0.162) |  | 0.0% |
| Lung function |  |  |  |  |  |
| median FVC>85% and median DLco>60% | 4 | 182 | 0.066 (0.035, 0.106) | 0.944 | 11.3% |
| median FVC≤85% or median DLco≤60% | 4 | 47 | 0.063 (0.013, 0.145) |  | 0.0% |

Abbreviations: N: number of patients; ES: effect size; FVC: forced vital capacity; DLco: diffusing capacity of the lungs for carbon monoxide.

**Supplemental Table 6. NSCLC patients versus** **NSCLC-ILD patients.**

|  | **NSCLC** | **N** | **NSCLC-ILD** | **N** | **P Values** |
| --- | --- | --- | --- | --- | --- |
| **CB+nab-PTX** |  |  |  |  |  |
| ORR | 33% | 521 | 43% | 684 | <0.05 |
| **CB+PTX** |  |  |  |  |  |
| ORR | 25% | 531 | 43% | 684 | <0.05 |
| **DDP+PEM** |  |  |  |  |  |
| ORR | 30.6% | 839 | 43% | 684 | <0.05 |
| 1-yOS | 43.5% | 839 | 33% | 481 | <0.05 |
| **DDP+GEM** |  |  |  |  |  |
| ORR | 28.2% | 830 | 43% | 684 | <0.05 |
| 1-yOS | 41.9% | 830 | 33% | 481 | <0.05 |

Abbreviations: N: number of patients; CB: carboplatin; DDP: cisplatin; nab-PTX: nano albumin paclitaxel; PTX: paclitaxel; PEM: pemetrexed; GEM: gemcitabine.

**Supplemental Figure 1. A funnel plot of acute exacerbation of interstitial lung disease (AE-ILD) rate.**


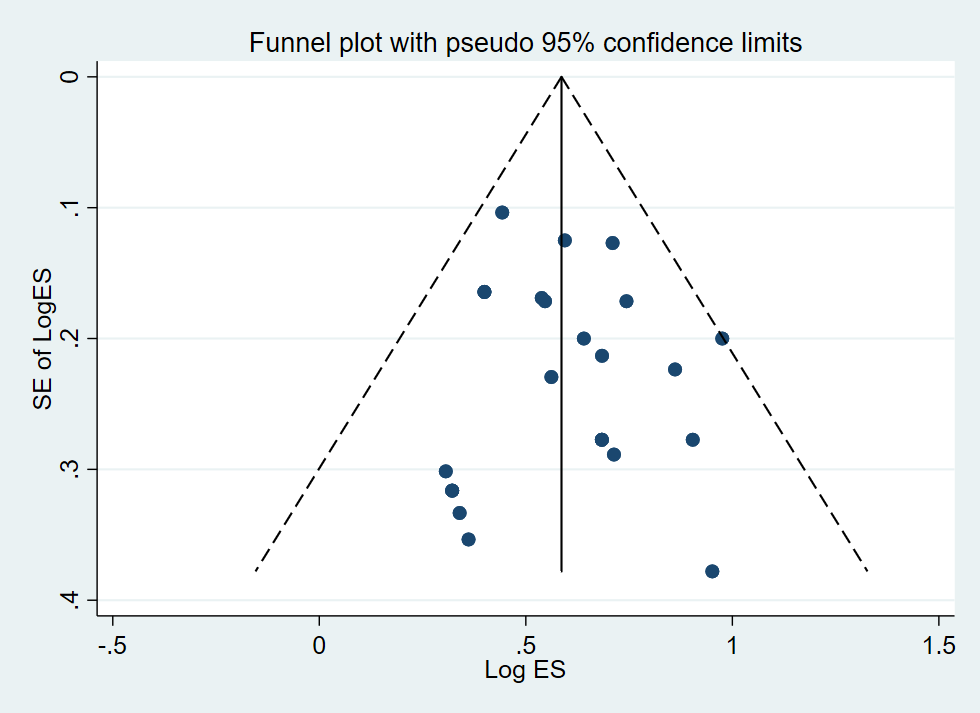


**Supplemental Figure 2. Subgroup analysis of objective response rate (ORR) of chemotherapy for patients with non-small cell lung cancer and interstitial lung disease (NSCLC-ILD).**


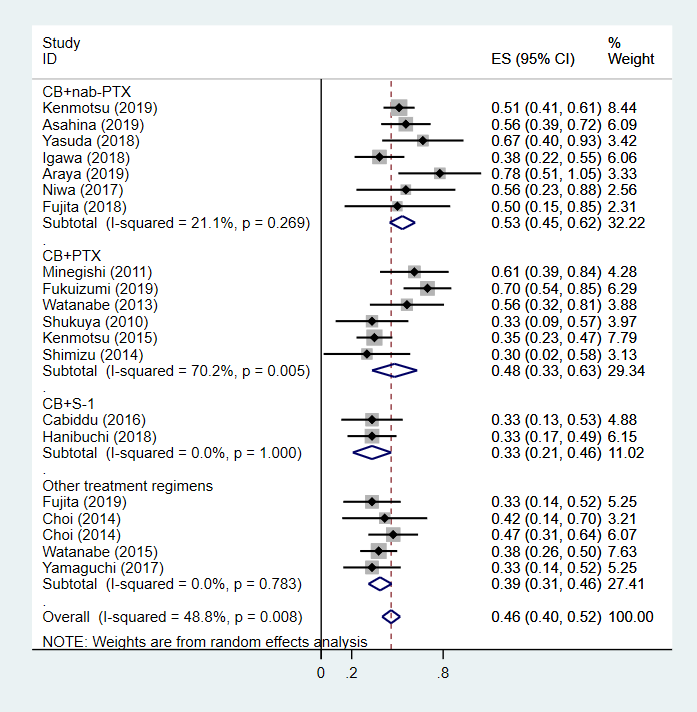


**Supplemental Figure 3. The pooled disease control rate (DCR) of chemotherapy for patients with non-small cell lung cancer and interstitial lung disease (NSCLC-ILD).**


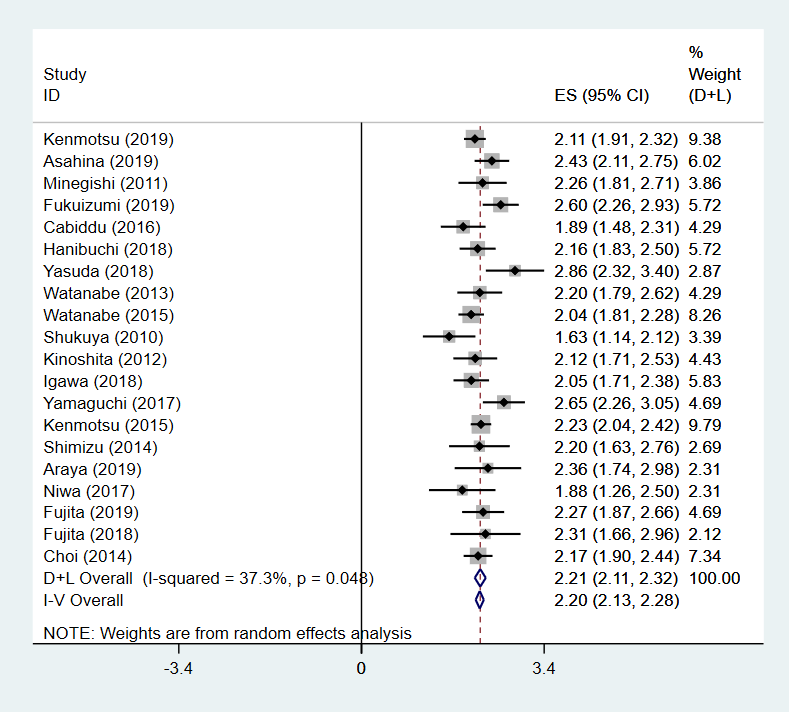


**Supplemental Figure 4. The pooled 1-year overall survival (1-yOS ) rate of chemotherapy for patients with non-small cell lung cancer and interstitial lung disease after omitting two studies.**

**
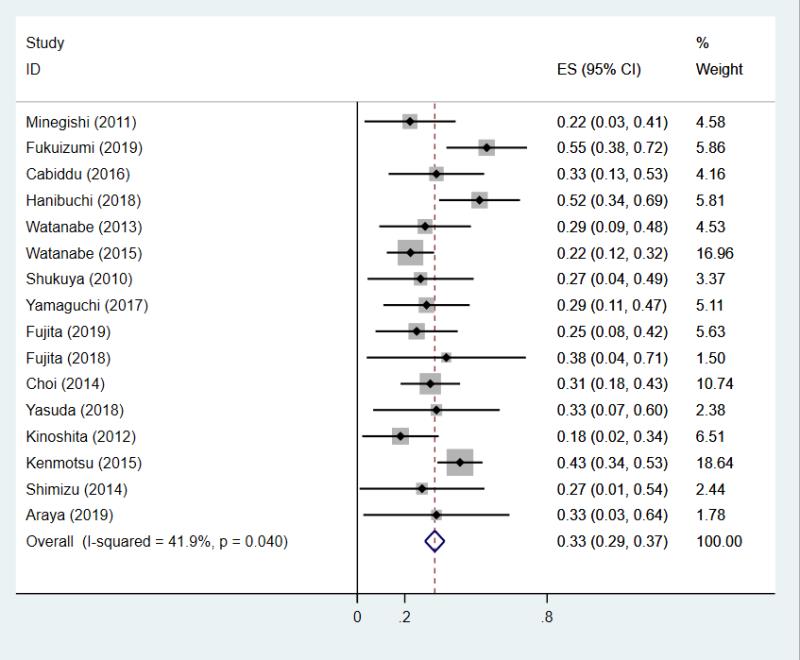
**

**Supplemental Figure 5. Subgroup analysis of acute exacerbation of interstitial lung disease (AE-ILD) rate of chemotherapy for patients with non-small cell lung cancer and interstitial lung disease (NSCLC-ILD).**


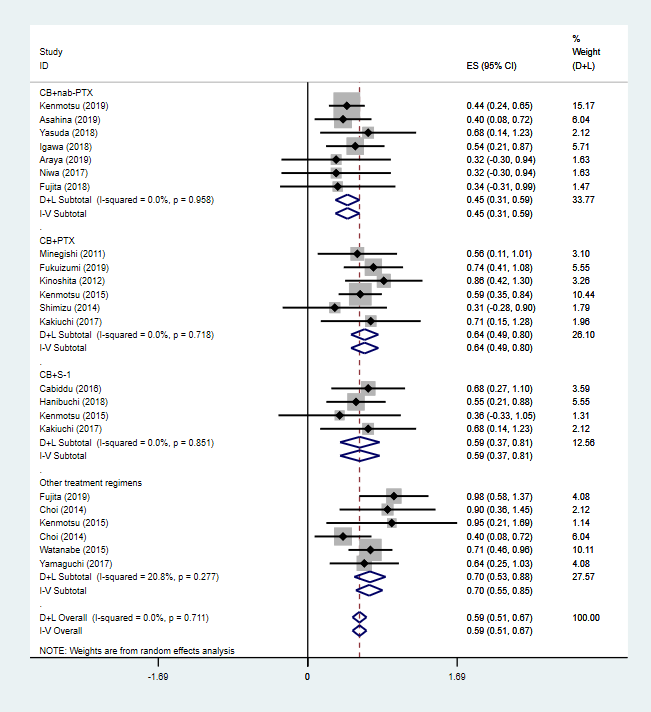

Supplement: Supplementary file 1 [file Data_Sheet_1.docx]
